# World AMR Awareness Week 2026: Raising Awareness and Promoting Behavioral Changes

**DOI:** 10.56786/PHWR.2026.19.43.1

**Published:** 2026-11-12

**Authors:** Yeo Won Jin, Eun Young Choi, Young-Il Jeong, In Kyoung Kim, U Jin Cho, Na-Ri Shin

**Affiliations:** Division of Antimicrobial Resistance Control, Department of Healthcare Safety and Immunization, Korea Disease Control and Prevention Agency Cheongju Korea

**Keywords:** Antimicrobial resistance (AMR), World AMR Awareness Week (WAAW), National action plan on AMR, Public awareness-raising activities

## Abstract

**Objectives:**

The Quadripartite organizations working on antimicrobial resistance (AMR) organize World AMR Awareness Week (WAAW), a global campaign to raise AMR awareness and encourage action across sectors. This article reviews recent developments and the role of WAAW in raising AMR awareness, presents the theme of WAAW 2026, and introduces the Korea Disease Control and Prevention Agency’s (KDCA) awareness-raising activities proposed for WAAW 2026.

**Methods:**

We reviewed the literature on WAAW and analyzed national survey findings on AMR awareness in the Republic of Korea (ROK), along with policy directions outlined in the Third National Action Plan on AMR (2026–2030).

**Results:**

WAAW, launched by the World Health Organization (WHO) in 2015, is a global campaign held annually from November 18 to 24. It is now coordinated jointly by the Quadripartite organizations within a multisectoral framework that encompasses human, animal, plant, and environmental health. Analysis of WAAW 2024 highlighted its role in raising global awareness of AMR through engagement with diverse stakeholders. WAAW 2026 will be organized using the theme, “One Health, one action: Prevent AMR now.” Meanwhile, sustaining public and media attention and reaching diverse audiences remain key challenges for WAAW. Moreover, in the ROK, efforts to raise AMR awareness are being pursued under the Third National Action Plan on AMR (2026–2030), with an emphasis on behavior change.

**Conclusions:**

The KDCA will strengthen targeted education and outreach activities in collaboration with relevant ministries and partners, emphasizing practical actions that individuals and communities can take to prevent AMR.

**Key messages**

**① What is known previously?**

Combating antimicrobial resistance (AMR) requires responsible antibiotic use and behavioral changes. World AMR Awareness Week (WAAW), promoted by the Quadripartite organizations, mobilizes global efforts to raise awareness and encourage action against AMR.

**② What new information is presented?**

Although WAAW engages stakeholders worldwide, sustaining public and media attention and reaching diverse audiences remain ongoing challenges. The 2026 campaign emphasizes the One Health approach, highlighting the need for coordinated action across sectors.

**③ What are implications?**

During WAAW 2026, the Korea Disease Control and Prevention Agency will strengthen targeted education and outreach in collaboration with relevant ministries and partners. These efforts aim to translate AMR awareness into action by promoting practical steps that individuals and communicates can take to prevent AMR.

## The Need to Respond to Antimicrobial Resistance (AMR) and Improve Awareness

AMR, particularly bacterial AMR, is a major global public health threat that impairs the effectiveness of treating and preventing bacterial infectious diseases [1]. The spread of AMR increases the risk of disease incidence and mortality, threatens the safety of essential medical procedures such as surgery and cancer treatment, and imposes a burden on the overall healthcare system through increased medical costs [2]. This issue of AMR is no longer a future threat, but a public health crisis that requires a response now. The World Health Organization (WHO) projects that more than 4.7 million deaths related to antimicrobial-resistant bacteria occur annually, and if urgent action is not taken, cumulative deaths directly attributable to antimicrobial-resistant bacteria could reach up to 39 million between 2025 and 2050. Furthermore, it is expected that the AMR crisis will cause annual losses of over 3.4 trillion USD to the global economy, having a greater impact particularly on low-income countries [3,4].

In response to the AMR crisis, WHO adopted the “Global Action Plan on Antimicrobial Resistance” in 2015 and proposed improving AMR awareness through effective communication, education, and training as the first of its five strategic objectives [5]. Improving awareness is important because it can induce appropriate behavior changes among various stakeholders involved in antibiotic use, thereby contributing to reducing the occurrence and spread of AMR. In 2015, WHO launched World AMR Awareness Week (WAAW) during one week in November as a global campaign to improve AMR awareness; the following year, in 2016, it was strengthened into a jointly operated system based on a tripartite collaboration involving the Food and Agriculture Organization of the United Nations and the World Organisation for Animal Health. Subsequently, the United Nations Environment Programme joined the Tripartite organizations to expand them into the Quadripartite organizations, and since 2023, it has been operated as a global campaign from a One Health perspective encompassing humans, animals, plants, and the environment. In addition, since 2020, the campaign period has been set annually from November 18 to 24, selecting a theme that contains key directions for responding to AMR and conducting campaigns to promote awareness and behavior change in the international community centered around this theme [3,6]. In other words, WAAW is an international campaign where the world intensively participates in awareness-raising activities under a common theme during a set period, alongside continuous efforts for AMR prevention and management.

## Recent Trends in WAAW and the 2026 Outreach Direction of the Korea Disease Control and Prevention Agency

WAAW is operated around a campaign theme selected to reflect major current issues and the flow of international discussions in responding to AMR, raising awareness and urging action in the international community regarding the theme (Table 1). The 2024 WAAW campaign was conducted under the theme “Educate. Advocate. Act now.”, going beyond education and raising awareness regarding AMR to call for policy support and concrete action [7]. Through this approach, the participation of a broader range of actors than in the past, such as patient groups, civil society, and youth, was expanded, and a global campaign was conducted using various media and methods, including mass media, social networking services (SNS), and online and offline events. The dedicated website for WAAW 2024 was visited by a total of 51,600 users from September 15 to December 31, 2024, of which 23,400 were recorded as new users. During the same period, total page views on the website reached 99,600. During the campaign period, the hashtag “#AntimicrobialResistance” recorded approximately 10,800 posts and 143,000 user reactions, showing that while the volume of mentions decreased compared to the previous year, the level of user engagement increased significantly. In addition, in 2024, through the “AMR is invisible. I am not.” campaign, content using lived experiences of AMR damage was produced and disseminated, recording over 2.7 million impressions and 51,537 user reactions on WHO SNSs [8]. These achievements demonstrate that WAAW functions not merely to raise awareness about AMR, but as an international awareness-raising campaign that expands the participation of various stakeholders. In 2025, under the theme “Act Now: Protect Our Present, Secure Our Future.”, joint action was emphasized to immediately respond to the current crisis of AMR and protect future health. Accordingly, concrete action and cross-sectoral collaboration were urged through discussions and webinars involving multi-sectoral stakeholders, activities for youth and students, and online participation campaigns.

The theme for WAAW 2026 is “One Health, one action: Prevent AMR now” (Figure 1). It urges individuals to start taking one action each to prevent AMR, emphasizing the interconnectedness of humans, animals, plants, food, and the environment [3]. In particular, at the 79th World Health Assembly in May 2026, the Global Action Plan on Antimicrobial Resistance 2026–2036, jointly prepared by the Quadripartite organizations on AMR, was adopted. The new action plan focuses on strengthening joint response across sectors based on a One Health approach that encompasses not only humans but also animals, plants, food, and the environment [2,9]. In line with this international trend, the Republic of Korea (ROK) is also strengthening its multi-sectoral integrated education and awareness campaign strategies to raise national awareness of AMR and drive behavior change. The Third National Action Plan on AMR (2026–2030) proposed strategies to expand target educational audiences to induce behavior change and promote year-round multi-sectoral integrated awareness-raising activities. The plan includes developing tailored educational materials considering the characteristics of target groups, such as adolescents, the general public, and the elderly and infirm, to apply them to various fields, including schools, hospitals, and households, while monitoring the effectiveness of education and outreach activities through regular awareness surveys of healthcare professionals and the public. In addition, year-round and target-specific awareness-raising campaigns connected with WAAW will be operated, and continuous education and outreach activities targeting various stakeholders will be promoted—such as strengthening education for stakeholders related to sectoral antibiotic use and resistance—aiming to achieve awareness-raising and behavior change goals by 2030 [10].

The the Korea Disease Control and Prevention Agency (KDCA) has been promoting education and campaigns to improve AMR awareness for the public and adolescents using WAAW as an opportunity. During WAAW in 2026, these activities will focus even more on transitioning from awareness improvement to behavior change. First, seven relevant government ministries and agencies^1)^ participating in national AMR management will jointly produce and distribute AMR awareness posters to inform the public about sectoral AMR management activities and provide guidance on appropriate antibiotic use practices that can be adopted in daily life. In addition, in cooperation with the Ministry of Education and the Korean Health Teachers Association, home newsletters on AMR and appropriate antibiotic use will be distributed to primary, middle, and high school students and parents nationwide. Through this approach, the plan aims to support the formation of correct awareness and behavior regarding antibiotic use not only among students but also in households. Furthermore, considering that childhood and adolescence are crucial periods for forming health knowledge, attitudes, and behaviors, AMR prevention model schools will be operated, and an educational approach in which the school community—including students, parents, and teachers—can participate together will be used to raise awareness regarding appropriate antibiotic use. This strategy is expected to contribute to building a foundation where education and outreach activities for raising AMR awareness lead to actual behavior change in daily life. In addition, it is essential that appropriate use and management of antibiotics are practiced across humans, animals, plants, and the environment, such that antibiotic prescribers appropriately prescribe antibiotics, antibiotic users correctly take them according to prescription guidelines, and leftover antibiotics are safely discarded.

## Limitations and Areas for Improvement in AMR Awareness Campaigns

To sustain these education and promotional activities over the mid-to-long term and enhance their effectiveness, it is necessary to establish strategies that can effectively reach diverse target groups while continuously maintaining public interest. Despite the expansion of awareness-raising activities for AMR, limitations remain in drawing sustained public interest. One study showed that the internet search volume for AMR-related campaigns temporarily spiked every November since 2017. On the other hand, the search volume for “antimicrobial resistance” peaked between February and April every year, rather than in November when campaigns are concentrated, and no distinct upward trend was observed during the observation period. Furthermore, the average search volume for AMR-related campaigns was significantly lower than that for other major disease awareness campaigns, standing at approximately 1/8 of prostate cancer campaigns and about 1/219 of breast cancer campaigns [11]. Meanwhile, a study analyzing media attention to AMR also showed that the frequency of media mentions of AMR increased slightly from 2015 to 2017 before declining in 2018, and the increase in media coverage associated with WAAW was not consistently observed [12]. This finding suggests that more strategic promotion and resource allocation are required to maintain continuous interest from the public and the media in AMR and to effectively spread awareness-raising activities.

In addition, it is important to prepare tailored strategies for AMR awareness promotion to improve information accessibility across diverse social groups. An evaluation of four national AMR campaigns in the UK using the Health Equity Assessment Tool revealed that consideration for certain marginalized and vulnerable groups was limited. Accordingly, the research team suggested the need to continuously develop materials and resources through collaboration with diverse user groups and to continuously monitor health inequalities during campaign development and evaluation processes [13]. Therefore, to enhance the effectiveness of AMR awareness-raising activities, it is crucial to move beyond uniform promotion aimed at the general public and establish strategies that reduce gaps in information access by considering group-specific characteristics, such as age, language, disability, and socioeconomic status.

## From Awareness to Action: Actions to Overcome the AMR Crisis

In the ROK, despite antibiotic consumption remaining high—ranking second among OECD countries at 33.6 DID^2)^ in 2024—the level of awareness regarding AMR remains very low. According to the 2025 AMR Awareness Survey, only 22.6% of respondents accurately knew that antibiotics are used to treat bacterial infections, while 72.0% incorrectly believed that antibiotics help treat the common cold [14]. Therefore, it is necessary to promote continuous and systematic education and public awareness campaigns to foster correct awareness of AMR and lead to appropriate antibiotic use behaviors. In fact, a study analyzing AMR awareness campaigns conducted worldwide reported cases showing significant improvements not only in knowledge and attitudes but also in antibiotic prescribing, usage, and behavior change [15]. This finding demonstrates that antibiotic education and public awareness campaigns do not merely stop at increasing knowledge and awareness of AMR but can also contribute to inducing appropriate antibiotic use behaviors.

AMR is a complex issue that is difficult to resolve with the single message, “antibiotic use must be reduced.” From the One Health perspective, which recognizes that humans, animals, plants, food, and the environment are interconnected, it is important to communicate the roles and responsibilities of each sector together. Going a step further from raising awareness about the severity of AMR, WAAW 2026 should serve as an opportunity to present specific actions that the public can practice in their respective positions. KDCA, in cooperation with relevant ministries and other relevant parties, plans to continuously strengthen targeted education and outreach activities so that correct awareness regarding AMR can lead to action.

## Figures and Tables

**Figure 1:**
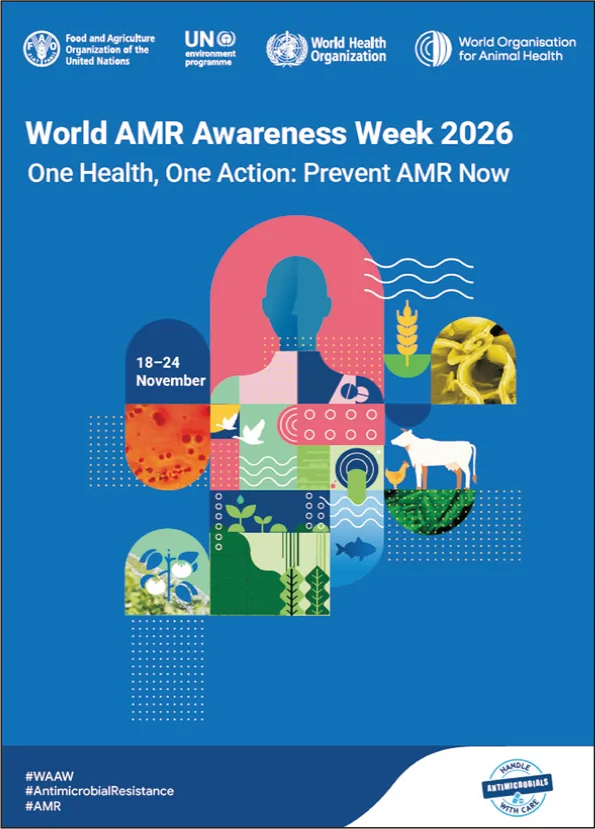
World AMR Awareness Week campaign poster Data from World Health Organization [3]. AMR=antimicrobial resistance.

**Table 1:** World AMR Awareness Week campaign themes by year, 2015–2026

Year	Campaign theme
2015	Antibiotics: Handle with Care
2016	Antibiotics: Handle with Care
2017	Seek advice from a qualified healthcare professional before taking antibiotics
2018	Change can’t wait. Our time with antibiotics is running out
2019	The future of antibiotics depends on all of us
2020	Handle with care: United to preserve antimicrobials
2021	Spread Awareness, Stop Resistance
2022	Preventing antimicrobial resistance together
2023	Preventing antimicrobial resistance together
2024	Educate. Advocate. Act now.
2025	Act Now: Protect Our Present, Secure Our Future
2026	One Health, one action: Prevent AMR now

AMR=antimicrobial resistance.

## Footnotes

1) Ministry of Health and Welfare, Ministry of Agriculture, Food and Rural Affairs, Ministry of Climate, Energy and Environment, Ministry of Oceans and Fisheries, Ministry of Food and Drug Safety, Rural Development Administration, Korea Disease Control and Prevention Agency.

2) DID: Refers to how many DDDs (Defined Daily Dose, the average maintenance dose per day for an adult) were consumed per 1,000 inhabitants per day.
