# Supplementary material for: World AMR Awareness Week 2026: Raising Awareness and Promoting Behavioral Changes
Source: Public Health Wkly Rep. 2026 Nov 12;19(43):1789–801. doi: 10.56786/PHWR.2026.19.43.1 (PMC13759226; doi:10.56786/PHWR.2026.19.43.1)
Supplement: Translation

# Translation: 2026년 세계 항생제 내성 인식주간: 인식개선과 행동 변화

**Authors:** 여원 진, 은영 최, 영일 정, 인경 김, 유진 조, 나리 신

**Affiliations:** 질병관리청 의료안전예방국 항생제내성관리과

**Keywords:** 항생제 내성, 세계 항생제 내성 인식주간, 국가 항생제 내성 관리대책, 인식개선 홍보

## Abstract

**목적:**

항생제 내성 국제 4자 협력기구는 항생제 내성 인식 제고를 위한 글로벌 캠페인을 운영하고 있다. 본 글에서는 글로벌 캠페인의 최근 동향과 의의를 살펴보고, 2026년 항생제 내성 글로벌 캠페인의 주제와 이에 따른 질병관리청의 인식개선 홍보 방향을 소개하고자 하였다.

**방법:**

항생제 내성 인식개선을 위한 글로벌 캠페인 결과와 영향에 대한 문헌 고찰과 국내 인식 수준 및 「제3차 국가 항생제 내성 관리대책(2026–2030)」에 따른 국가 정책 방향 등을 종합적으로 분석하였다.

**결과:**

2015년 세계보건기구에 의해 최초 시작된 세계 항생제 내성 인식주간(World AMR Awareness Week, WAAW)은 11월 18일부터 24일까지 시행되는 글로벌 캠페인으로, 최근 인간‧동물‧식물‧환경을 포괄하는 국제 4자 협력기구의 공동 운영으로 확대되었다. 2024년 WAAW는 다양한 이해관계자의 참여를 확대하는 국제적 인식개선 캠페인으로서의 역할이 확인되었고, 2026년 WAAW는 “One Health, one action: Prevent AMR now”를 주제로 진행될 예정이다. 한편, 항생제 내성 캠페인은 대중과 언론의 지속적인 관심을 유지하고 다양한 계층에 효과적으로 도달하는 것이 중요한 과제로 제시되었으며, 우리나라는 「제3차 국가 항생제 내성 관리대책(2026–2030)」을 기반으로 인식 제고와 행동 변화를 위한 국가 차원의 교육‧홍보를 강화하고 있다.

**결론:**

질병관리청은 2026년 WAAW 핵심 주제의 취지를 적극 반영하여 관계 부처 등과 함께 국민이 각자의 자리에서 실천할 수 있는 구체적인 행동을 제시하는 대상별 교육과 홍보를 집중적으로 실시할 예정이다.

**핵심요약**

**① 이전에 알려진 내용은?**

항생제 내성에 효과적으로 대응하기 위해서는 올바른 항생제 사용과 예방 행동이 중요하며, 국제 4자 협력기구는 대중의 인식 제고를 위한 글로벌 캠페인을 운영하고 있다.

**② 새로이 알게 된 내용은?**

세계 항생제 내성 인식주간(World AMR Awareness Week, WAAW)은 다양한 이해관계자가 참여하는 국제적 캠페인으로 기능하고 있다. 또한 대중과 언론의 지속적인 관심을 유지하고 다양한 계층에 효과적으로 도달하는 것이 중요한 과제로 제시되었다. 2026년 WAAW는 One Health 접근을 강조하며 분야 간 공동 대응과 구체적인 실천을 촉구하고 있다.

**③ 시사점은?**

우리나라의 항생제 내성 인식개선과 행동 변화를 위해서는 2026년 WAAW 기간에 단순한 정보 제공을 넘어 대상별로 이해하기 쉽고 일상에서 실천할 수 있는 구체적인 행동수칙을 제시하는 교육‧홍보가 중요하다.

## 항생제 내성 대응과 인식개선의 필요성

항생제 내성(antimicrobial resistance), 특히 세균성 항생제 내성은 세균 질환 감염 치료 및 예방 효과를 저해하는 중요한 전 세계적 공중보건 위협 중 하나이다[1]. 항생제 내성의 확산은 질병 발생과 사망 위험을 증가시키고, 수술‧항암 치료 등 필수적인 의료행위의 안전성을 위협하며, 의료비 증가 등을 통해 보건의료시스템 전반에 부담을 초래한다[2]. 이러한 항생제 내성 문제는 더 이상 미래의 위협이 아니라 현재 대응이 필요한 공중보건 위기이다. 세계보건기구(World Health Organization, WHO)는 항생제 내성균 관련 사망이 매년 470만 명 이상 발생하고, 긴급한 조치가 취해지지 않으면 항생제 내성균에 직접 기인한 사망의 경우 2025년부터 2050년까지 누적 최대 3,900만 명에 이를 것으로 전망한다. 또한 항생제 내성 위기는 세계 경제에 연간 3조 4천억 달러 이상의 손실을 초래하고, 특히 소득이 낮은 국가에 더 큰 영향을 미칠 것으로 예상한다[3,4].

항생제 내성 위기에 대응하여 WHO는 2015년에 「항생제 내성에 관한 글로벌 행동 계획(Global Action Plan on Antimicrobial Resistance)」을 채택하고 효과적인 소통‧교육‧훈련을 통한 항생제 내성 인식 향상을 5대 전략 중 첫 번째 전략으로 제시하였다[5]. 인식개선이 중요한 이유는 항생제 사용과 관련된 다양한 주체의 적절한 행동 변화를 유도하고, 이를 통해 항생제 내성의 발생과 확산을 줄이는 데 기여할 수 있기 때문이다. 2015년 당시 WHO는 항생제 내성 인식개선을 위한 글로벌 캠페인으로 11월 한 주간 세계 항생제 내성 인식주간(World AMR Awareness Week, WAAW) 운영을 시작하였고, 이듬해인 2016년부터는 유엔식량농업기구(Food and Agriculture Organization of the United Nations)와 세계동물보건기구(World Organisation for Animal Health; 당시 Office International des Épizooties)가 참여하는 3자 협력기구 기반의 공동 운영 체계로 강화되었다. 이후 3자 협력기구에 유엔환경계획(United Nations Environment Programme)이 합류하여 4자 협력기구로 확대되면서, 2023년부터는 인간‧동물‧식물‧환경을 포괄하는 One Health 관점의 글로벌 캠페인으로 운영되고 있다. 또한 2020년부터는 매년 11월 18일부터 24일까지를 캠페인 기간으로 정하여, 항생제 내성 대응의 주요 방향을 담은 주제를 선정하고 이를 중심으로 국제사회의 인식 제고와 행동 변화를 촉진하는 캠페인을 전개하고 있다[3,6]. 즉, WAAW는 항생제 내성 예방‧관리를 위한 지속적인 노력과 더불어, 정해진 기간에 전 세계가 공동의 주제 아래 인식개선 활동에 집중적으로 동참하는 국제 캠페인이다.

## WAAW의 최근 동향과 질병관리청의 2026년 홍보 방향

WAAW는 항생제 내성 대응의 주요 현안과 국제적 논의의 흐름을 반영하여 선정된 캠페인 주제를 중심으로 운영되며, 해당 주제에 대한 국제사회의 인식을 높이고 실천을 촉구하고 있다(표 1). 2024년 WAAW는 “Educate. Advocate. Act now.”를 주제로 항생제 내성에 대한 교육과 인식 제고를 넘어 정책적 지지와 구체적인 행동을 촉구하는 방향으로 추진되었다[7]. 이를 통해 환자단체, 시민사회, 청년 등 과거보다 더 광범위한 주체의 참여를 확대하고 대중매체, 사회관계망서비스(social networking services, SNS), 온‧오프라인 행사 등 다양한 매체와 방식을 활용하여 전 세계적인 캠페인을 전개하였다. WAAW 2024 전용 누리집은 2024년 9월 15일부터 12월 31일까지 전체 이용자 51,600명이 방문하였으며, 그중 신규 이용자는 23,400명으로 집계되었다. 같은 기간 누리집의 페이지 조회 수는 총 99,600회에 달했다. 캠페인 기간 중 ‘#AntimicrobialResistance’ 해시태그는 약 10,800건의 게시물과 143,000건의 이용자 반응을 기록하여, 전년도보다 언급량은 감소했으나 이용자 반응 수준은 크게 증가한 것으로 나타났다. 또한 2024년에는 ‘AMR is invisible. I am not.’ 캠페인을 통해 항생제 내성 피해 경험 사례를 활용한 콘텐츠를 제작‧확산하였으며, WHO SNS에서 270만 회 이상의 노출과 51,537건의 이용자 반응을 기록하였다[8]. 이러한 성과는 WAAW가 항생제 내성에 대한 인식을 높이는 데 그치지 않고 다양한 이해관계자의 참여를 확대하는 국제적 인식개선 캠페인으로 기능하고 있음을 보여준다. 2025년에는 “Act Now: Protect Our Present, Secure Our Future.”를 주제로 항생제 내성의 현재 위기에 즉각 대응하고 미래의 건강을 보호하기 위한 공동의 행동을 강조하였다. 이에 따라 여러 분야 이해관계자가 함께 참여하는 토론과 웨비나, 청년‧학생 대상 활동, 온라인 참여 캠페인 등을 통해 구체적인 행동과 분야 간 협력을 촉구하였다.

2026년 WAAW의 주제는 “One Health, one action: Prevent AMR now”이다(그림 1). 사람‧동물‧식물‧식품‧환경이 서로 연결되어 있다는 점을 강조하면서, 항균제 내성 예방을 위해 각자가 실천할 수 있는 행동을 하나씩 시작할 것을 촉구하는 내용이다[3]. 특히 2026년 5월 제79차 세계보건총회에서는 항생제 내성 국제 4자 협력기구가 함께 마련한 「항생제 내성에 관한 글로벌 행동 계획 2026–2036」이 채택되었다. 새 행동 계획은 사람뿐만 아니라 동물‧식물‧식품‧환경을 포괄하는 One Health 접근을 바탕으로 각 분야의 공동 대응을 강화하는 데 초점을 두고 있다[2,9]. 우리나라도 이러한 국제적 흐름에 발맞춰 국가 차원의 항생제 내성 인식 제고와 행동 변화를 위한 다부문 통합 교육‧홍보 전략을 강화하고 있다. 「제3차 국가 항생제 내성 관리대책(2026–2030)」에서는 행동 변화를 유도하기 위해 교육 대상을 확대하고, 연중 다부문 통합 인식개선 홍보를 추진하는 전략을 제시하였다. 청소년, 일반인, 노약자 등 대상별 특성을 고려한 맞춤형 교육자료를 개발하여 학교, 병원, 가정 등 다양한 현장에 적용하고, 의료인과 국민 등에 대한 정기적인 인식도 조사를 통해 교육‧홍보의 효과를 모니터링할 계획이다. 또한 연중 및 WAAW와 연계한 대국민‧대상별 인식개선 캠페인을 운영하고, 부문별 항생제 사용 및 내성 관련 이해관계자에 대한 교육을 강화하는 등 다양한 주체를 대상으로 지속적인 교육‧홍보를 추진하여 2030년까지 인식 제고와 행동 변화 목표를 달성하기 위해 노력할 것이다[10].

질병관리청은 그동안 WAAW를 계기로 국민과 청소년 등을 대상으로 항생제 내성 인식개선을 위한 교육‧홍보를 추진해 왔다. 2026년 WAAW 기간에는 이러한 활동을 인식개선에서 행동 변화로의 전환에 더욱 중점을 둘 예정이다. 먼저 국가 항생제 내성 관리에 참여하는 7개 관계 부처^1)^가 함께 항생제 내성 관련 홍보 포스터를 제작‧배포하여 국민에게 분야별 항생제 내성 관리 활동을 알리고, 일상에서 실천할 수 있는 올바른 항생제 사용 행동을 안내할 계획이다. 또한 교육부 및 보건교사회와 협력하여 전국 초‧중‧고등학교 학생과 학부모를 대상으로 항생제 내성 및 올바른 항생제 사용에 관한 가정통신문을 배포할 예정이다. 이를 통해 학생뿐만 아니라 가정에서도 항생제 사용에 대한 올바른 인식과 행동이 형성될 수 있도록 지원할 계획이다. 아울러 아동‧청소년기는 건강에 대한 지식과 태도, 행동이 형성되는 중요한 시기라는 점을 고려하여 항생제 내성 예방 시범학교를 운영할 예정이며 학생, 학부모, 교사 등 학교 공동체가 함께 참여할 수 있는 교육적 접근을 통해 올바른 항생제 사용에 대한 인식을 높일 계획이다. 이는 항생제 내성 인식 제고를 위한 교육‧홍보가 실제 생활 속 행동 변화로 이어지는 기반 마련에 기여할 것으로 기대된다. 아울러 항생제 처방권자는 항생제를 적절하게 처방하고, 항생제 사용자는 처방 지침에 따라 올바르게 복용하며, 남은 항생제를 안전하게 폐기하는 등 사람‧동물‧식물‧환경 전반에서 항생제의 적절한 사용과 관리가 실천되도록 하는 것이 중요하다.

## 항생제 내성 인식개선 홍보 활동의 한계와 개선 과제

이러한 교육‧홍보 활동을 중장기적으로 지속하고 효과를 높이기 위해서는 대중의 관심을 지속적으로 유지하는 동시에 다양한 계층에 효과적으로 도달할 수 있는 전략을 마련할 필요가 있다. 항생제 내성에 대한 인식개선 활동이 확대되고 있음에도 불구하고, 대중의 지속적인 관심을 이끌어 내는 데에는 여전히 한계가 있다. 한 연구에서는 항생제 내성 관련 캠페인에 대한 인터넷 검색량이 2017년 이후 매년 11월에 일시적으로 증가하는 양상을 보였다. 반면 ‘항생제 내성’에 대한 검색량은 캠페인이 집중되는 11월이 아닌 매년 2–4월에 정점을 보였으며, 관측기간 동안 뚜렷한 증가 추세도 나타나지 않았다. 또한 항생제 내성 관련 캠페인의 평균 검색량은 다른 주요 질환 인식 캠페인에 비해 현저히 낮아, 전립선암 캠페인의 약 1/8, 유방암 캠페인의 약 1/219 수준으로 나타났다[11]. 한편, 항생제 내성에 대한 언론의 관심을 분석한 연구에서도 언론에서 항생제 내성이 언급되는 빈도가 2015–2017년 일부 증가한 이후 2018년에는 감소하는 양상을 보였으며, WAAW에 따른 언론 보도 증가도 일관되게 나타나지 않았다[12]. 이는 항생제 내성에 대한 대중과 언론의 지속적인 관심을 유지하고 인식개선 활동을 효과적으로 확산하기 위해 보다 전략적인 홍보와 자원 투입이 필요함을 시사한다.

그리고 항생제 내성 인식개선 홍보는 다양한 계층의 정보 접근성을 높일 수 있도록 맞춤형 전략을 마련하는 것도 중요하다. 영국의 국가 단위 항생제 내성 캠페인 4개를 건강 형평성 평가도구(Health Equity Assessment Tool)를 활용하여 평가한 결과, 일부 소외‧취약 집단에 대한 고려가 제한적인 것으로 확인되었다. 이에 연구진은 다양한 이용자 집단과의 협력을 통해 자료와 자원을 지속적으로 개발하고, 캠페인 개발 및 평가 과정에서 건강 불평등을 지속적으로 점검할 필요가 있다고 제안하였다[13]. 따라서 항생제 내성 인식개선 활동의 효과를 높이기 위해서는 전체 대중을 대상으로 한 일률적인 홍보를 넘어, 연령‧언어‧장애‧사회경제적 여건 등 집단별 특성을 고려하여 정보 접근의 격차를 줄일 수 있는 전략을 마련하는 것이 중요하다.

## 인식에서 행동으로: 항생제 내성 위기 극복을 위한 실천

우리나라는 항생제 사용량이 2024년 33.6 DID^2)^로 OECD 국가 중 2위에 위치할 정도로 높은 수준임에도 불구하고, 항생제 내성에 대한 인식 수준은 여전히 매우 낮은 상황이다. 2025년 항생제 내성 인식도 조사에 따르면 항생제가 세균 감염 치료에 사용된다는 사실을 정확하게 알고 있는 응답자는 22.6%에 그쳤으며, 항생제가 감기 치료에 도움이 된다고 잘못 알고 있는 응답자는 72.0%에 달했다[14]. 따라서 항생제 내성에 대한 올바른 인식을 형성하고 적정한 항생제 사용 행동으로 이어지도록 지속적이고 체계적인 교육‧홍보를 추진할 필요가 있다. 실제로 전 세계에서 시행된 항생제 내성 인식개선 캠페인을 분석한 연구에서는 지식과 태도 개선뿐만 아니라 항생제 처방‧사용 및 행동 변화에서도 유의한 개선이 나타난 사례가 보고되었다[15]. 이는 항생제 교육‧홍보가 항생제 내성에 대한 지식과 인식을 높이는 데 그치지 않고, 올바른 항생제 사용 행동을 유도하는 데에도 기여할 수 있음을 보여준다.

항생제 내성은 ‘항생제 사용을 줄여야 한다’는 하나의 메시지만으로 해결하기 어려운 문제이다. 사람‧동물‧식물‧식품‧환경이 서로 연결되어 있다는 One Health 관점에서 각 분야의 역할과 책임을 함께 알리는 것이 중요하다. 2026년 WAAW는 항생제 내성의 심각성을 알리는 데서 한 걸음 더 나아가 국민이 각자의 자리에서 실천할 수 있는 구체적인 행동을 제시하는 계기가 되어야 한다. 질병관리청은 관계 부처 등과 협력하여 항생제 내성에 대한 올바른 인식이 실천으로 이어질 수 있도록 대상별 교육과 홍보를 지속적으로 강화해 나갈 예정이다.

## Figures and Tables

**그림 1:** 2026년 세계 항생제 내성 인식주간 캠페인 포스터 Data from World Health Organization [3]. AMR=antimicrobial resistance.

**표 1:** 연도별 세계 항생제 내성 인식주간 캠페인 주제(2015–2026년)

연도	캠페인 주제
2015	Antibiotics: Handle with Care
2016	Antibiotics: Handle with Care
2017	Seek advice from a qualified healthcare professional before taking antibiotics
2018	Change can’t wait. Our time with antibiotics is running out
2019	The future of antibiotics depends on all of us
2020	Handle with care: United to preserve antimicrobials
2021	Spread Awareness, Stop Resistance
2022	Preventing antimicrobial resistance together
2023	Preventing antimicrobial resistance together
2024	Educate. Advocate. Act now.
2025	Act Now: Protect Our Present, Secure Our Future
2026	One Health, one action: Prevent AMR now

AMR=antimicrobial resistance.

## Footnotes

1) 보건복지부, 농림축산식품부, 기후에너지환경부, 해양수산부, 식품의약품안전처, 농촌진흥청, 질병관리청

2) DID: 인구 1,000명 당 하루에 얼마의 DDD (Defined Daily Dose, 성인이 하루 동안 복용해야 하는 평균 유지 용량)를 소비했는지 의미
